# Changes in Crystal Structure and Accelerated Hydrolytic Degradation of Polylactic Acid in High Humidity

**DOI:** 10.3390/polym13244324

**Published:** 2021-12-10

**Authors:** Yutaka Kobayashi, Tsubasa Ueda, Akira Ishigami, Hiroshi Ito

**Affiliations:** 1 Research Center for GREEN Materials and Advanced Processing (GMAP), 4-3-16 Jonan, Yonezawa 992-8510, Japan; akira.ishigami@yz.yamagata-u.ac.jp; 2 Graduate School of Organic Materials Science, Yamagata University, 4-3-16 Jonan, Yonezawa 992-8510, Japan; t211686d@st.yamagata-u.ac.jp

**Keywords:** biopolymers, hydrolytic degradation, crystal structure

## Abstract

Highly crystallized polylactic acid (PLA) is suitable for industrial applications due to its stiffness, heat resistance, and dimensional stability. However, crystal lamellae in PLA products might delay PLA decomposition in the environment. This study clarifies how the initial crystal structure influences the hydrolytic degradation of PLA under accelerated conditions. Crystallized PLA was prepared by annealing amorphous PLA at a specific temperature under reduced pressure. Specimens with varied crystal structure were kept at 70 °C and in a relative humidity (RH) of 95% for a specific time. Changes in crystal structure were analyzed using differential calorimetry and wide-angle X-lay diffraction. The molecular weight (MW) was measured with gel permeation chromatography. The crystallinity of the amorphous PLA became the same as that of the initially annealed PLA within one hour at 70 °C and 95% RH. The MW of the amorphous PLA decreased faster even though the crystallinity was similar during the accelerated degradation. The low MW chains of the amorphous PLA tended to decrease faster, although changes in the MW distribution suggested random scission of the molecular chains for initially crystallized PLA. The concentrations of chain ends and impurities, which catalyze hydrolysis, in the amorphous region were considered to be different in the initial crystallization. The crystallinity alone does not determine the speed of hydrolysis.

## 1. Introduction

Polylactic acid (PLA), as a bio-polymer, is expected to assist the transition to a carbon-neutral world. A low-molecular-weight (MW) PLA was prepared by ring-opening polymerization in 1932, and a high-MW PLA was made from purified raw materials in 1954 [1]. Moreover, in 1954, polypropylene (PP) was discovered [2]. The global production capacity of PLA in 2020 was 390,000 tons [3], while that of PP was 90 million tons [4]. One of the reasons for such a large difference is the performance of polymeric material.

The goal of PLA researchers has been to achieve long-term durability, molding processability, and thermal stability. Thus, the optical purity of the L-form has been increased, the polymerized product has been purified, and the chain ends of the polymer have been protected [5]. Currently, high-performance PLA with high crystallinity is manufactured. However, the improvement in durability indicates a decrease in degradability in the environment. Hence, PLA has caused plastics waste. In particular, PLA is not marine degradable [6].

After a long period in the ocean, PLA becomes a microplastic, similar to PP. The environmental impact of microplastics can be divided into the absorption of toxic chemicals and their own toxicity. Since PLA is less hydrophobic, it adsorbs fewer polynuclear aromatics [7], but the biological activity of the sediments made from microplastics may affect organisms [8]. Thus, there is a demand for a plastic with high marine decomposition properties. Not limited to synthetic or natural polymers, highly degradable plastics are characterized by a low glass transition temperature (Tg), low fusion heat, and low hydrophobicity [9]. It is difficult to achieve both degradability and mechanical performance.

Molded products of amorphous PLA will be deformed above the Tg of 65 °C. To stabilize the dimensions of products at high temperatures, sufficiently crystallized PLA is utilized. Factors that control the crystallinity of PLA are the processing conditions, the optical purity of polymer chains, and doped additives such as plasticizers and nucleating agents. Crystallized PLA shows that enzymatic degradation occurs at the surface of the products, the amorphous part is lost, and lamella crystals remain during surface erosion [10]. The same surface erosion occurs in an alkaline aqueous solution [11]. The hydrolysis reaction is unlikely to occur inside the crystal.

On the other hand, the hydrolysis of PLA in a neutral solution proceeds with a bulk erosion mechanism, in which water molecules diffuse inside the molded product. The permeability coefficient of water vapor at 25 °C decreases by half as the crystallinity of PLA increases [12]. However, with the hydrolysis of crystallized PLA in a 37 °C buffer solution, little depends on the differences in crystallinity [13]. In some cases, the PLA with greater crystallinity decomposes faster than the amorphous PLA [14], as there could be a different factor than the permeability coefficient that could be measured over a shorter time.

The development of marine-degradable materials requires accelerated testing of durability such as hydrolysis to shorten the evaluation time. Generally, the Arrhenius plot estimates the durability of polymer materials [15]. PLA crystallizes rapidly at Tg and above the temperature in hydrolysis tests [16], although crystallization progresses gradually near room temperature [13]. It is important in accelerated tests that the hydrolysis of molecular chains and changes in the crystal structure are accelerated similarly. In this study, we investigated the effect of the initial crystal structure on the hydrolysis of PLA in a high-temperature and high-humidity environment. In particular, the relationships between the internal morphology, crystallinity, and mechanical properties of molded PLA products were analyzed at different annealing temperatures and levels of humidity.

## 2. Materials and Methods

A Total Corbion (Rayong, Thailand) L175 with a melt mass flow rate (MFR) of 3 g/10 min at 190 °C was used as the PLA. The film samples were prepared using a compression-molding machine manufactured by Imoto Seisakusho (Kyoto, Japan). The PLA pellets were pressurized at 10 MPa for 2 min after heating and melting at 190 °C for 4 min under reduced pressure; then, they were cooled at 20 °C with a pressure of 10 MPa for 2 min. The film thickness was about 150 μm. To change the crystal structure, The PLA films were annealed at a predetermined temperature for 2 h in an AS ONE (Osaka, Japan) AW-250N vacuum oven. The constant temperature and humidity treatment of the sample were carried out at 70 °C and 95% relative humidity (RH) for a predetermined time using Tokyo Rika Kikai (Tokyo, Japan) KCL-2000W.

The tensile test was carried out using a Sanko (Nagoya, Japan) ISL-T300 at a span of 18 mm and a tensile speed of 1 mm/min at room temperature. The dumbbell sample was prepared by punching a JIS No. 7 dumbbell from a PLA sheet at room temperature. The crystal morphology inside the sheet was observed using an Olympus (Tokyo, Japan) BX-51P polarized optical microscope. A 20 μm-thick film was cut from the cross-section of the sheet using a sliding microtome Leica (Tokyo, Japan) RM2125. An impregnating solution was added to the preparation.

**Figure 1:**
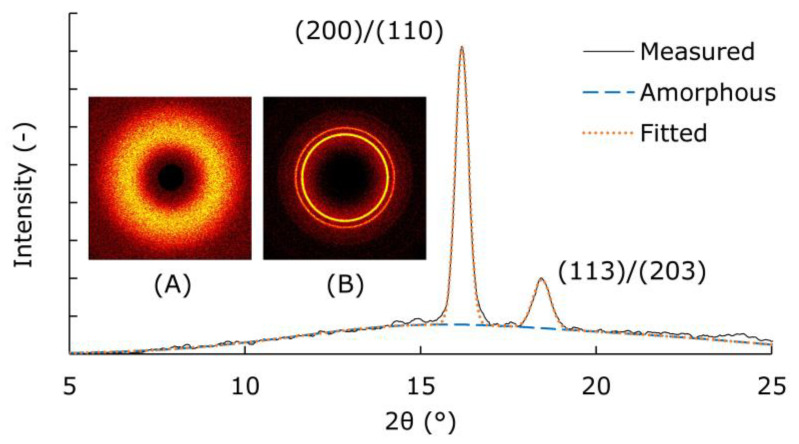
One-dimensional (1D) profile of wide-angle X-ray diffraction of the annealed PLA at 110 °C under reduced pressure. Amorphous halo (**A**); diffraction from (200), (110), (203), and (113) planes (**B**).

The crystal structure of the PLA was analyzed by wide-angle X-ray diffraction (WAXD) using Rigaku (Tokyo, Japan) Smart Lab. The measurement conditions were as follows: X-ray, Cu (Kα); camera length, 28.5 mm; exposure time, 10 min; and the detector was a HyPix-3000. Three samples were sandwiched between the Kapton tapes (3M Japan, Tokyo, Japan) for measurement. From the measured 2D image, the background including Kapton tape was corrected using SmartLab Studio II software (Rigaku, Tokyo, Japan). Figure 1 shows how the crystallinity was calculated using Igor Pro 6.0 software (WaveMetrics, Portland, OR, USA). The integral value of the amorphous halo (A) in the azimuth direction was fitted by a Gaussian function to obtain an amorphous 1D profile. From the 1D profile of the semi-crystalline sample (B), the crystal planes (200) (110) and (113) (203) were fitted by a Gaussian function to obtain the area ratio of crystals to an amorphous halo.

**Figure 2:**
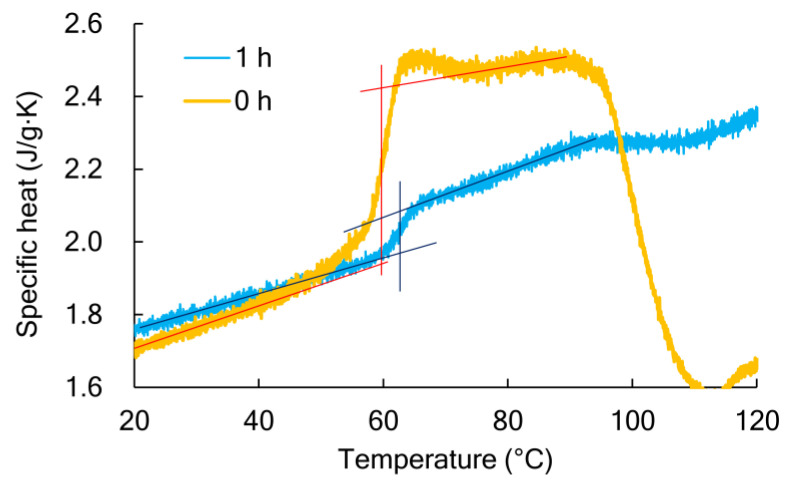
Comparison of specific heat between the amorphous PLA (0 h) and annealed PLA at 70 °C and 95% RH (1 h).

Thermal characterization was performed by differential scanning calorimetry (DSC) using the TA Instruments (New Castle, DE, USA) Q200. The samples were kept at room temperature for 24 h under reduced pressure before DSC measurement. The temperature was raised from 0 to 200 °C at 10 °C/min and the glass transition, cold crystallization, and melting behavior were measured. The crystallinity was calculated with the heat of fusion of the perfect crystal as 143 J/g [17]. The mobile amorphous portion was quantified from the ratio of the change in specific heat (ΔC_p_) at the glass transition temperature to the completely amorphous ΔC_p_ 0.531 J g^−1^K^−1^ [18]. Figure 2 shows the change in specific heat near Tg. The tangent line was extended to Tg from the specific heat of the glass state on the low-temperature side and the rubber state on the high-temperature side, and the difference was defined as ΔC_p_.

The MW of the PLA was determined by a gel permeation chromatography (GPC) using a Waters Corporation 515 HPCL pump, 2414 RI, and Agilent Technologies PLgel 5μ MID XED-C columns. The solvent was chloroform. The measurement temperature was 40 °C and the PS conversion method was used for the MW calibration. The number average molecular weight (Mn) of Total Corbion L175 was 99,600 Da, and the weight average molecular weight (Mw) was 217,000 Da. This measurement is comparable to the previously reported results [19].

## 3. Results

### 3.1. Crystal Structure of Annealed PLA

In this study, we observed changes in the crystal structure that occurred during the test at 70 °C and 95% RH using samples with varied initial crystallinities. To change the crystal structure, the compression-molded PLA film was annealed under predetermined conditions. Table 1 shows the annealing temperature, crystallinity (χ_c_), and tensile properties. The reference sample (STD) rapidly cooled at 20 °C was confirmed by wide-angle X-ray diffraction to be in an amorphous state with no out-of-plane orientation (Figure 1A). This STD was annealed at a predetermined temperature for 2 h under reduced pressure. Although annealing was performed at a temperature higher than the Tg of 65 °C, almost no crystallization occurred at 80 °C. The crystallinity was approximately 30% for the samples annealed from 90 to 110 °C. The crystallinity increased with the annealing, and the tensile strain at break decreased.

**Figure 3:**
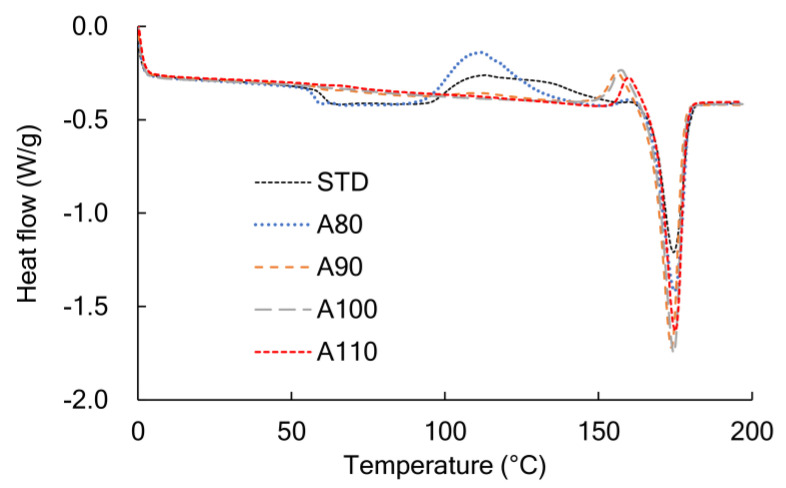
DSC curves during heating at 10 °C/min for the annealed PLA.

Annealing PLA changes not only the crystallinity but also the polymorphism. A DSC curve measured in a heating process included the information of rigid amorphous, α-form crystals, and α’-form crystals. In Figure 3, the STD and A80 showed a Tg around 60 °C, and the heat of cold crystallization (Tcc) around 120 °C. The peak of Tcc was apparently shifted to a lower temperature by annealing [20]. For A90, A100, and A110, a transition from α’-form to α-form crystal appeared at around 160 °C instead of the Tcc [21,22]. Thus, the amorphous molecular chains changed to an α’-form crystal during annealing at less than 110 °C under reduced pressure.

### 3.2. Influence of Accelerated Environment on Crystal Structure

**Figure 4:**
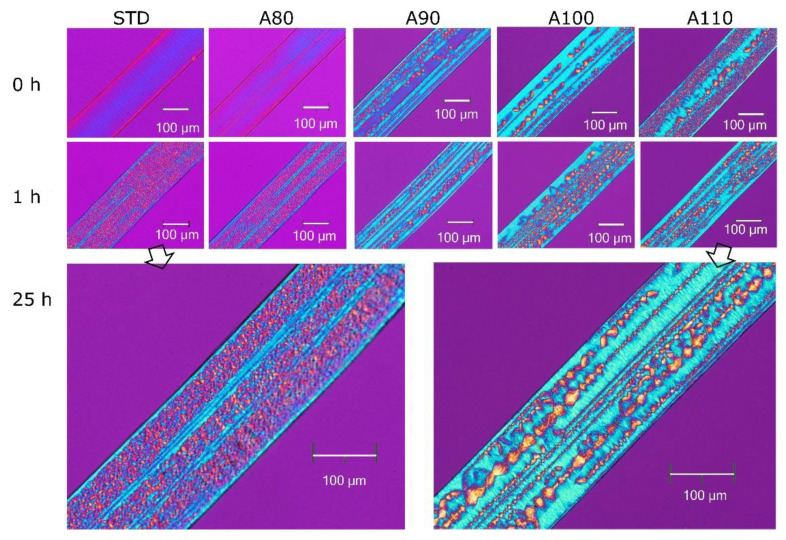
Changes in crystal morphology observed with POM during hydrolysis at 70 °C and 95% RH for PLA annealed at various temperatures.

Annealed PLA specimens were placed in a chamber at 70 °C and 95% RH for a specific length of time. Then, changes in the crystal structure were measured with POM and DSC. Figure 4 shows the POM micrographs of the cross-section of the specimens after a predetermined time had passed. Specimens at 0 h, which meant initially annealed, changed significantly depending on whether the annealing temperature was 80 °C or less or 90 °C or more. Birefringence was observed in the cross-section of the STD; the molecular chains were in-plane oriented despite the amorphous scattering by WAXD. The annealed specimens A90, A100, and A110 showed oriented crystals and spherulites. This corresponded to the crystallinity shown in Table 1.

After 1 h at 70 °C and 95% RH, the STD and A80 changed from amorphous to spherulite structures. On the other hand, the crystal morphology produced by annealing had little change at the same conditions. Even after 25 h in the chamber, there was little visual change in the crystal morphology. Thus, the initially amorphous PLA crystallized within 1 h. The crystallization rate of the hydrated PLA was higher than that in the dry state due to the increased level of molecular mobility [23]. The annealed PLA did not show the same morphology as the STD after 25 h—that is, the spherulitic morphology was formed from the amorphous PLA and not from the initially crystallized specimens. The molecular chains incorporated into the crystals indicate that they could not move freely even when hydrated.

**Figure 5:**
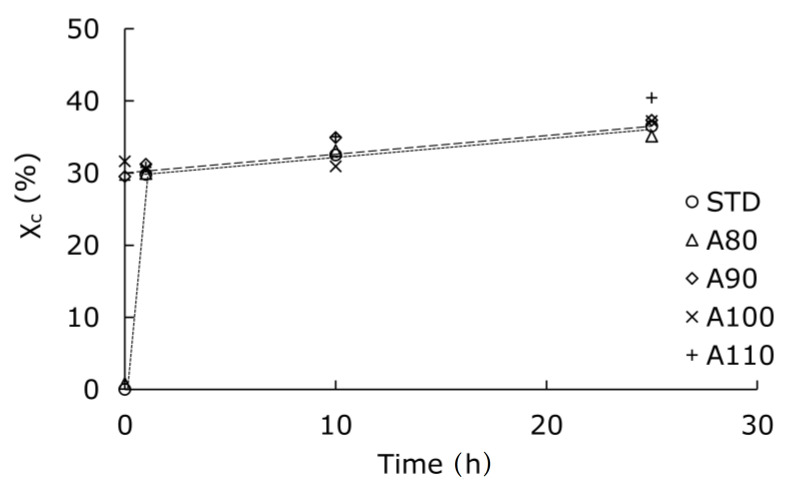
Changes in crystallinity during hydrolysis at 70 °C and 95% RH for PLA annealed at various temperatures.

Figure 5 shows the crystallinity measured by the DSC to quantitatively understand the change in the crystal structure. The horizontal axis represents the time for the accelerated test at 70 °C and 95% RH. The initially amorphous PLA reached a crystallinity of 30% in one hour. After 1 h or more, the crystallinity was similar regardless of the initial crystal structure. The crystal morphology shown in Figure 4 was different in the initial annealing conditions even though the crystallinity was similar. Although crystallization within 1 h is important, it was difficult to do the accelerated test in a short time due to a transient time of approximately 5 min for stabilizing the chamber. The time it takes for amorphous PLA to reach a crystallinity of 30% might be even shorter.

### 3.3. Changes in Tensile Fracture after the Accelerated Test

**Figure 6:**
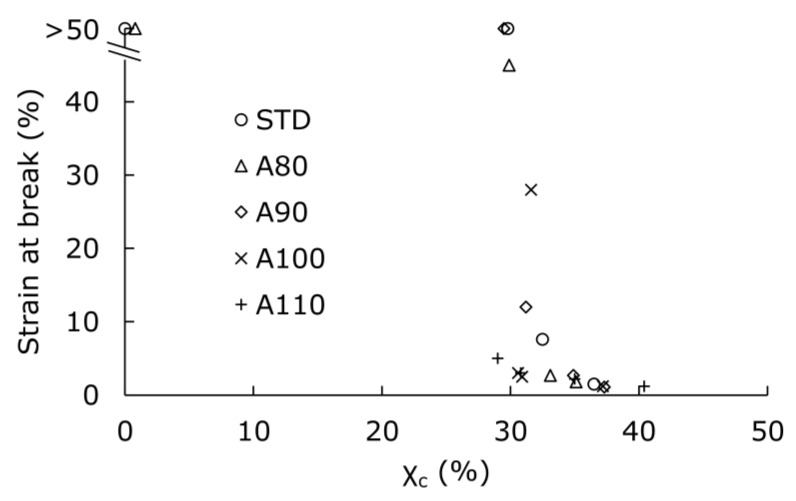
The relationship between crystallinity and strain at break for PLA annealed and hydrolyzed in predetermined conditions.

Generally, crystal morphology is considered to affect the physical properties of specimens. In this study, we focused on the change in strain at breaking point in the tensile tests. Figure 6 shows the relationship between crystallinity and strain at break. Regardless of the initial crystal state, when the crystallinity reached 30% after the accelerated test, the strain at break sharply decreased. Increasing crystallinity generally reduces the strain at break. However, even with similar crystallinity, some stretched and some fractured. Thus, the critical reason for the difference in elongation was examined.

**Figure 7:**
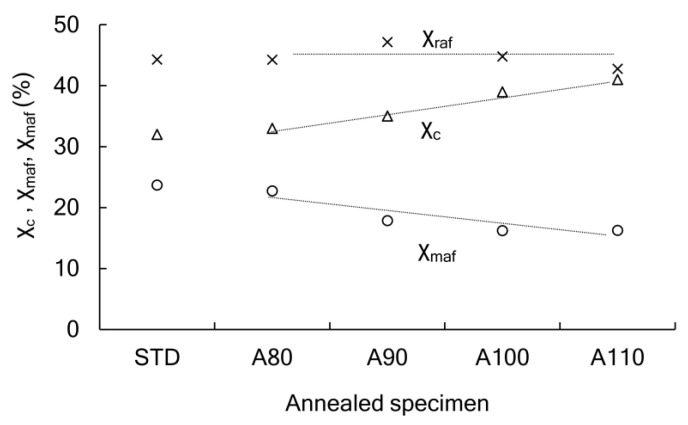
Ratio of crystal (χ_c_), mobile amorphous (χ_maf_), and rigid amorphous (χ_raf_) for annealed PLA after the 1 h hydrolysis at 70 °C and 95% RH.

**Figure 8:**
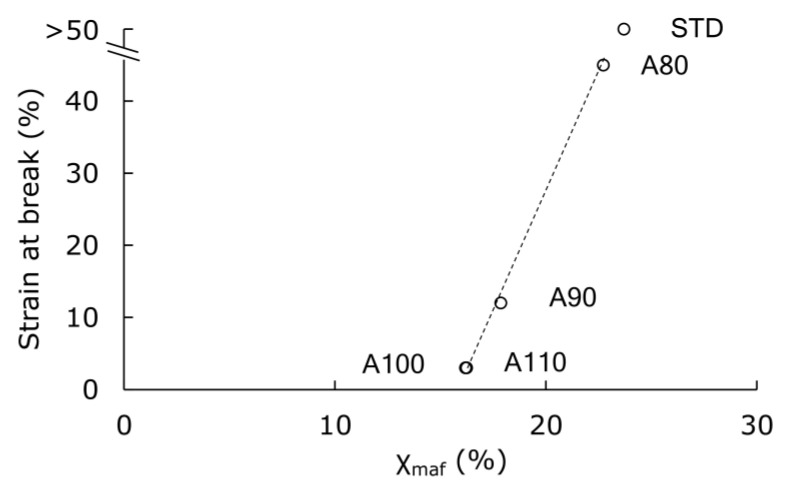
The relationship between mobile amorphous (χ_maf_) and strain at break for annealed PLA after the 1 h hydrolysis at 70 °C and 95% RH.

The index of higher-order structure includes not only crystallinity but also rigid amorphous. Although the crystallinity measured using DSC was the same in the specimens set for 1 h at 70 °C and 95% RH, the detailed structures were analyzed again. The WAXD analysis was added, and we attempted to separate the amorphous sections into mobile amorphous (χ_maf_) and rigid amorphous (χ_raf_) groups using the gap in specific heat change at Tg. Figure 7 shows the ratio of χ_c_ χ_maf_ χ_raf_ for each specimen. The comparison of the STD to the A110 suggested that the initial annealing increased χ_c_ and decreased χ_maf_. There was little change in χ_raf_. Thus, we focused on the amount of χ_maf_. As shown in Figure 8, strain at break increased when χ_maf_ was 17% or more. Since the tensile test was performed at a room temperature lower than Tg, the amount of χ_maf_, which indicated higher molecular mobility, affected the elongation of specimens.

### 3.4. Changes in Molecular Weight Distribution after Accelerated Test

**Figure 9:**
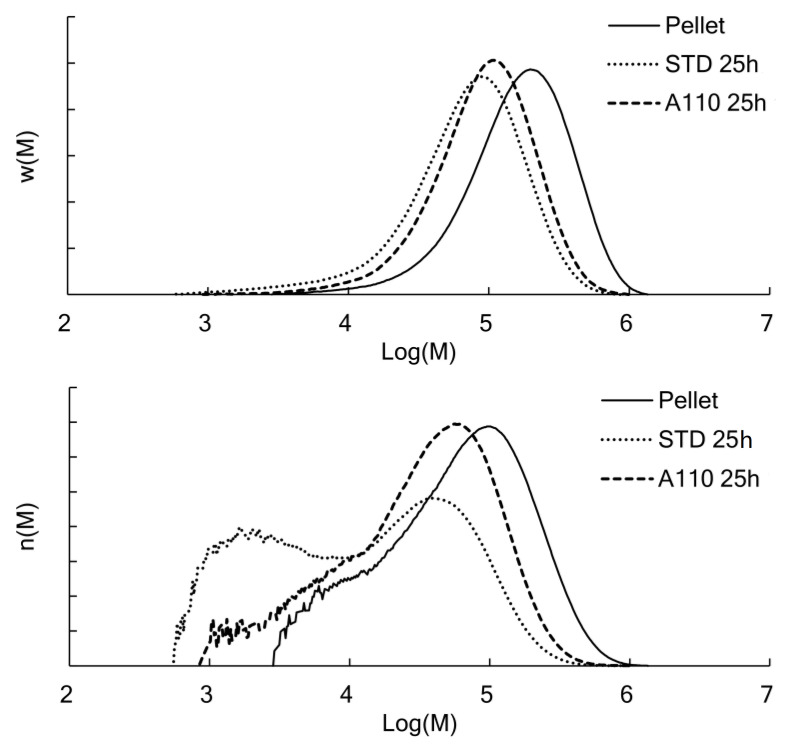
GPC curves with vertical axes of weight fraction (w(M)) and molar fraction (n(W)). Comparison of the raw pellet and the amorphous and annealed specimens hydrolyzed for 25 h.

As described above, each specimen had comparable crystallinity except during the first hour. We examined whether the initial crystal structure affected hydrolysis for the remaining 24 h. Figure 9 shows the changes in the molecular weight distribution (MWD) of PLA due to hydrolysis. The MWD of PLA after the 25 h test was compared to that of raw pellets with no processing history as a reference. The Mn decreased from 217 kDa in Pellet to 98.1 kDa in STD 25 h and 122 kDa in A110 25 h. The polydispersity was 2.2, 3.1, and 2.2, respectively. The difference in the polydispersity was that the MW peak of A110 25 h shifted toward a lower MW, as shown in both curves of weight fraction w(M) and mole fraction n(M). On the other hand, in STD 25 h, the low-molecular-weight component increased remarkably.

In the decomposition of polymer chains, the asymptotic value of polydispersity is two as the secession probability of each repeating unit is equal—that is, random secession occurs [24]. Therefore, the molecular chains were randomly cleaved, although A110 was in a semi-crystalline state, where the mobility of chains was considered to be different in amorphous and crystal phases. On the other hand, the STD indicated that the low MW portion had become even lower in MWD.

## 4. Discussion and Conclusions

The hydrolysis of PLA often proceeds at temperatures below its Tg in the natural environment. In contrast, accelerated degradation at a high temperature and humidity is generally performed in the laboratory. In researching the relationship between the crystal structure of molded PLA products and their hydrolysis, there was concern that the difference in crystal structure between the initial specimens would be lost during the accelerated test. In this experiment as well, the amorphous PLA reached a crystallinity of 30% within 1 h at 70 °C and 95% RH—that is, the difference in crystallinity of the initially annealed specimens disappeared. However, as shown in Figure 9, the initial crystal structure greatly affected the decrease in MW.

There are two possible reasons for the difference in the MWD change by hydrolysis between STD and A110. First, the STD was hydrolyzed during the crystallizing amorphous process. Since hydrolysis is likely to proceed in the amorphous state, some molecular chains were cleaved before the crystals were sufficiently grown within 1 h at 70 °C and 95% RH, as shown in Figure 5. The low MW portion was further reduced in MW as a catalytic effect of the terminal carboxyl group of the cleaved molecular chains being concentrated in the amorphous region in the process of crystallization.

Second, the terminal groups and impurities, which promote the decomposition of polymer chains, are not as concentrated in the amorphous region in the process of A110 crystallization under reduced pressure. As shown in Figure 4, the crystal morphology of A110 is different to that of the STD crystallized in the presence of water. The STD has a fine spherulite structure due to its high molecular mobility with moisture. Thus, the diffusion of terminal groups and impurities is different in the moisture of crystallization conditions.

The hierarchy of these two reasons for accelerated hydrolysis in STD is still unknown—that is why the low MW portion is further reduced in MW. In contrast, the random decrease in MW shown by A110 indicates that it is possible to produce an industrial product that decomposes without distinguishing between crystalline and amorphous portions. Highly crystalline PLA, which has a high stiffness and thermal stability, might hydrolytically degrade without becoming microplastics made of lamellar crystals if the crystallization process is controlled. This is an industrially important finding.

## Figures and Tables

**Table 1:** Characteristics of PLA annealed at predetermined temperatures.

	Annealing	χ_c_ *	Tensile Properties		
	Temperature		Modulus	Strength	Strain at Break
	(°C)	(%)	(MPa)	(MPa)	(%)
STD	-	-	2730	57.9	>50
A80	80	1	3370	69.0	>50
A90	90	30	3270	71.0	>50
A100	100	32	3520	69.6	28
A110	110	29	3300	68.0	5

* DSC method.

## Data Availability

The data presented in this study are available on request from the corresponding author.
